# Nonsurgical Retreatment of a 16 × 9 mm Osteolytic Lesion With Buccal Bone Regeneration Using a Bioceramic Sealer: A Clinical and NGS-Based Microbiological Case Report With 30-Month Follow-Up

**DOI:** 10.1155/crid/9703223

**Published:** 2025-10-09

**Authors:** Andrea Spinelli, Filippo d'Errico, Alessio Buonavoglia, Maria Giovanna Gandolfi, Carlo Prati, Fausto Zamparini

**Affiliations:** ^1^Endodontic Clinical Section, Dental School, Department of Biomedical and Neuromotor Sciences, University of Bologna, Bologna, Italy; ^2^Laboratory of Green Biomaterials and Oral Pathology, Dental School, Department of Biomedical and Neuromotor Sciences, University of Bologna, Bologna, Italy

**Keywords:** bioceramic sealer, calcium silicate sealer, CBCT, NGS, periapical lesion, root canal retreatment

## Abstract

This case report described the complete resolution of a large osteolytic lesion following a nonsurgical endodontic retreatment using a premixed bioceramic sealer. A 30-year-old healthy patient presented a large radiographic osteolytic lesion of 16 × 9 mm in correspondence with Teeth #42, #41, and #31. Clinical examinations revealed positive percussion tests and increased mobility of all incisors. The microbiological profile of the root canals was assessed through paper point sample collection, DNA extraction, and next-generation sequencing, revealing markedly different proportions of bacteria genera in the affected teeth. A secondary endodontic treatment was then performed, combining manual, rotary, and reciprocating protocols. A premixed bioceramic-based sealer (CeraSeal) was used for obturation due to the presence of wide and wet apexes. Radiographic (x-rays and CBCT) and clinical evaluations reported the progressive healing of the lesion over 30 months. This outcome supports the use of conservative treatments as a valid alternative to endodontic surgery for the management of extensive periapical lesions.

## 1. Introduction

Complications from trauma to the mandibular arch, resulting in periapical infection and large bone defects due to cyst formation, are relatively common. Traditionally, primary therapy involves surgical intervention to manage these extensive bone defects [1]. However, to mitigate the risks associated with invasive surgery, less invasive surgical approaches—marsupialization or decompression—can be employed [2, 3].

The histology of cystic lesions has been widely investigated, revealing the presence of different bacteria both inside and outside the root canal. Bacterial infections play an important role in the formation and growth of radicular cysts. The epithelial cells of Malassez proliferate in response to these infections, leading to cyst development. The inflammatory nature of these cysts highlights the importance of addressing bacterial infections to manage and treat these lesions effectively [4, 5].

Large periapical lesions, whether cystic or granulomatous, pose a significant challenge in endodontic treatment. Literature supports their management using a conservative approach with secondary root canal treatment, which is aimed at eliminating infection from the root canal system and promoting the healing of periapical tissues [6].

Premixed bioceramic-based sealers have become widely adopted in clinical practice for their bioactive properties associated with their ease of use [7]. These materials can promote osteogenesis and provide an optimal environment for the healing of periapical tissues [8–11]. Their biocompatibility makes premixed bioceramic sealers potentially the ideal choice for root canal obturation in the presence of large periapical lesions or necrotic teeth.

CeraSeal (Meta Biomed, South Korea) is a premixed bioceramic-based sealer composed of tricalcium silicate, dicalcium silicate, tricalcium aluminate, zirconium oxide, and calcium sulfate. In vitro studies have described its chemical and physical properties, highlighting its calcium ion release and alkalizing activity [12, 13]. Recent studies demonstrated a strong osteoinductive effect of this sealer on different populations of stem cells, such as periodontal ligament stem cells, osteoblasts, and vascular stem cells, suggesting a supportive role in inducing new bone formation in periapical bone defects [8, 14].

Next-generation sequencing (NGS) technologies have also become invaluable tools in endodontic research. This analysis offers detailed insights into the microbial communities present in infected root canals. By analyzing the 16S ribosomal RNA gene, NGS allows for the comprehensive identification of bacterial species, providing a deeper understanding of the microbial etiology of endodontic infections [15, 16]. The integration of advanced microbial analysis in clinical decision-making represents a novel approach that may benefit the endodontic community.

Despite the promising properties of premixed bioceramic-based sealers and the advanced capabilities of NGS, their combined application in the nonsurgical management of extensive periapical lesions is not widely documented in clinical literature. This case report is aimed at filling this gap by documenting a remarkable nonsurgical healing of a large periapical lesion using a premixed bioceramic sealer and detailing the microbial profile associated with the infection through NGS.

## 2. Case Report

**Figure 1:**
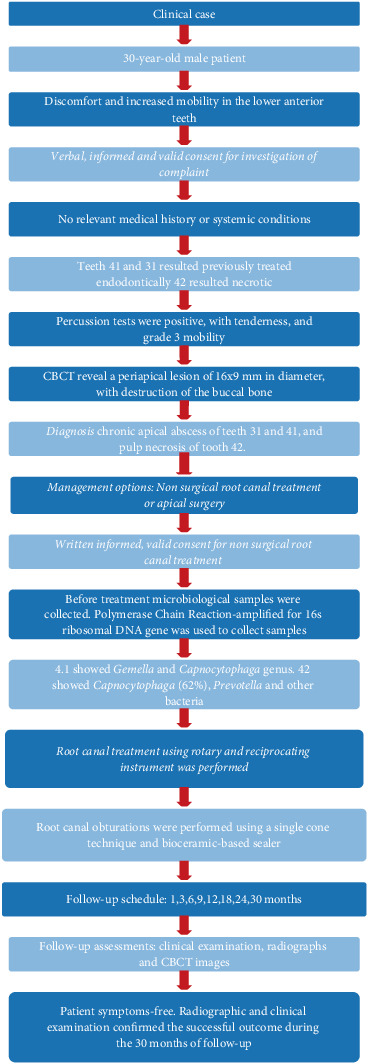
Flowchart of the case according to case report guidelines. From Nagendrababu V, Chong BS, McCabe P, Shah PK, Priya E, Jayaraman J, Pulikkotil SJ, Setzer FC, Sunde PT, Dummer PMH (2020) “PRICE 2020 Guidelines for Reporting Case Reports in Endodontics: A Consensus-Based Development.” *International Endodontic Journal* doi:10.1111/iej.13285. For further details, visit https://pride-endodonticguidelines.org/price/.

The manuscript is written in accordance with the Preferred Reporting Items for Case Reports in Endodontics (PRICE) guidelines [17]. The PRICE 2020 flowchart presents the case report summary (Figure 1). The completed PRICE 2020 checklist is provided as Figure S1.

The study was performed in agreement with the ethical guidelines of the Declaration of Helsinki laid down in 1964 and its later amendments or comparable ethical standards.

Ethical approval was obtained from the Ethics Committee of the Azienda Unità Sanitaria Locale of Bologna (Authorization No. 844-2021-OSS-AUSLBO-21160-ID). Written informed consent was obtained from participants prior to their inclusion in the study.

**Figure 2:**
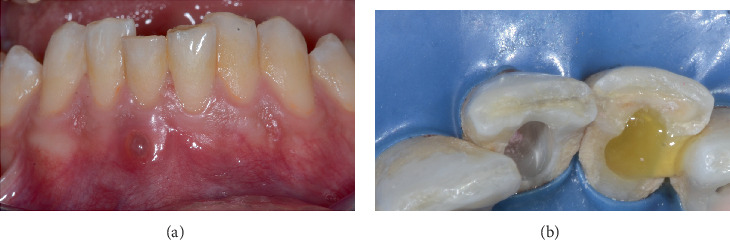
Clinical images of the fistula and pus. (a) Intraoral image showing the fistula associated with Tooth 41. The presence of a fistulous tract is visible in the vestibular region. (b) Clinical image showing pus discharge from Tooth 41, with yellowish purulent exudate observed spontaneously and upon applying pressure in the vestibular region.

**Figure 3:**
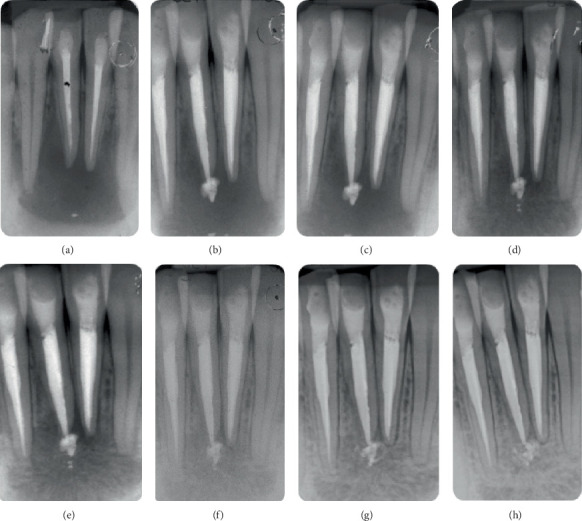
Timeline of radiographic evaluation of the periapical healing process. (a) Preoperative x-ray showing a huge periapical lesion involving Teeth 42, 41, and 31 with apical bone loss. (b) X-ray immediately after the endodontic treatments. Please note the periapical extrusion of the bioceramic sealer from Tooth 41. (c) X-ray at 3 months. The extrusion of the bioceramic sealer appears stable beyond the apex. (d) X-ray at 6 months showing initial bone healing and continued and stable presence of extruded sealer. (e) Radiograph at 12 months showing an improvement in bone regeneration and the initial integration of the extruded sealer into surrounding tissues. (f) Radiograph at 18 months with further signs of healing and no adverse reaction to the extruded sealer. (g) Radiograph at 24 months showing continued bone regeneration. (h) Radiograph at 30 months confirming complete resolution of the lesion, with almost full integration of the extruded sealer.

A 30-year-old healthy male was referred to our university department (Endodontic Clinical Section–Dental chool, University of Bologna) with a chief complaint of discomfort and increased mobility in the lower anterior teeth. The patient had no relevant medical history or systemic conditions. Clinical examination revealed swelling in the vestibular region of the mandibular anterior teeth, specifically Teeth 42, 41, and 31. Percussion tests were positive, indicating tenderness, and Grade 3 mobility of the affected teeth was noted. Vitality tests for Tooth 42 resulted negative. A fistulous tract was present in the vestibular region associated with Tooth 41 (Figure 2). Periapical radiographs revealed an osteolytic lesion affecting Teeth 42, 41, and 31 measuring approximately 16 × 9 mm (Figure 3). Teeth 41 and 31 had been previously treated endodontically.

**Figure 4:**
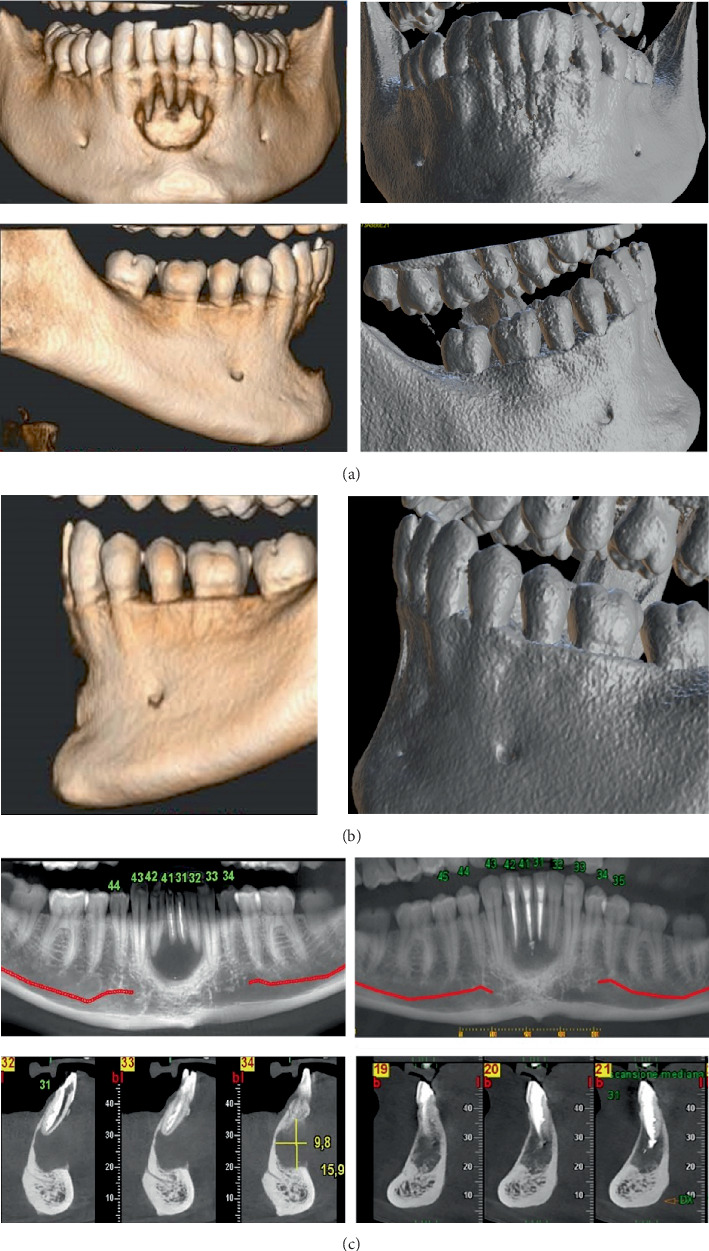
Preoperative and 6-month posttreatment CBCT analysis. (a) 3D reconstruction of the preoperative CBCT scan showing the large periapical lesion and cortical bone destruction and the 3D reconstruction at 6 months posttreatment demonstrating substantial buccal bone regeneration. (b) Axial sections of the CBCT scans pretreatment and posttreatment, highlighting the reduction in lesion size and the reformation of an initial bone structure. (c) Sagittal sections of the CBCT scans pretreatment and posttreatment providing a detailed view of the vestibular cortical bone regeneration.

A cone beam computed tomography (CBCT) was performed, revealing a large periapical lesion measuring 16 × 9 mm in diameter, associated with destruction of the buccal bone (Figure 4a). Based on these findings, the diagnosis was a chronic apical abscess of Teeth 31 and 41, characterized by the presence of a fistula and pulp necrosis of Tooth 42.

At the initial appointment, before prescribing antibiotics, microbiological samples were collected from Teeth 41 and 42 using sterile paper points inserted into the root canals. The patient was then prescribed antibiotics (amoxicillin and clavulanic acid, 875/125 mg, twice daily for 7 days). The treatment plan involved the retreatment of Teeth 41 and 31 and primary endodontic treatment of Tooth 42. During the diagnostic process, a surgical approach, such as an apicoectomy, was also considered. However, after discussing the options with the patient and taking into account his age, it was decided to proceed with a nonsurgical approach.

The patient agreed to the proposed treatment plan and signed the informed consent form after being fully informed about the methods, risks, and potential discomforts involved.

At the second appointment, 1 week later, the initial procedure included accessing the pulp chamber of Tooth 42 and the retreatment of Teeth 41 and 31. Spontaneous yellowish purulent exudate was observed from Tooth 41 (Figure 2b), which was further induced by applying pressure on the vestibular side. The working length was determined with an apex locator (Root ZX, Morita, Osaka, Japan) and confirmed through periapical radiographs. Root canal shaping was performed with rotary files up to size 25.06 (Rotate, VDW, Munich, Germany) to a working length of 25 mm for Tooth 42. A reciprocating instrument 25.08 (Reciproc Blue, VDW, München, Germany) was used to retreat Teeth 41 and 31. Abundant irrigation was performed using sodium hypochlorite solution (Niclor 5, OGNA, Muggiò, Italy). The access cavities were temporarily sealed with Coltosol (Coltene, Altstaetten, Switzerland).

At the third appointment, 1 week later, retreatment of Teeth 31 and 41 was continued. Reciproc Blue 50.05 was used to working lengths, followed by final apical instrumentation with K files 60.02. During the treatment, the exudate from Tooth 41 was drained, and abundant disinfection with saline solution alternated with sodium hypochlorite solution 5% was carried out. Temporary restorations were placed.

Two additional appointments were scheduled at 1-week intervals, specifically dedicated solely to rinses with sodium hypochlorite. Manual dynamic irrigation was used to enhance the efficacy of the irrigant solutions. After this, a saline solution was used. Finally, a 10% ethylenediaminetetraacetic acid (EDTA) solution was applied for the final irrigation.

After 1 month, we observed a complete resolution of the clinical symptoms, no tooth mobility, and the closure of the fistulous tract. Clinical examination revealed the absence of exudate, clean canals free from remnants of gutta-percha, and clear effluent of sodium hypochlorite from the canals.

Root canal obturations were performed using CeraSeal with cold single cone techniques and gutta-percha cones (Roeko Coltene/Whaledent, Altstatten, Switzerland) for all teeth. Postendodontic restorations were performed after 14 days using a two-step procedure. Clearfil SE Bond (Kuraray Co. Ltd, Osaka, Japan) was first applied, followed by a flowable composite on the dentine floor. The remaining portion was filled with G-ænial (GC Europe, Leuven, Belgium) composite resin.

Periodic clinical and radiographic follow-ups were conducted to monitor the healing process. Radiographs were taken at 3, 6, 12, 18, 24, and 30 months posttreatment (Figures 3b, 3c, 3d, 3e, 3f, 3g, and 3h). After 6 months, a CBCT scan confirmed the notable buccal bone regeneration and the initial healing process of the periapical lesion (Figures 4a, 4b, and 4c).

The radiographic and clinical examination confirmed the successful outcome during the 30 months of follow-up. The patient received regular professional hygiene maintenance, including scaling and polishing procedures, during this period (every 6 months).

The entire treatment was performed by two expert endodontists using an operating microscope (OMS3200 Dental Microscope, Zumax Medical Co., Suzhou, China).

### 2.1. Microbiological Findings

Microbiological analysis of samples from Teeth 41 and 42 was conducted using polymerase chain reaction amplification of the 16S ribosomal DNA gene and sequenced. The samples were collected during the first appointment, before antibiotic therapy, using sterile paper points inserted into the root canals of Teeth 41 and 42. Amplification for Tooth 31 was not possible due to the low bacterial load.

**Figure 5:**
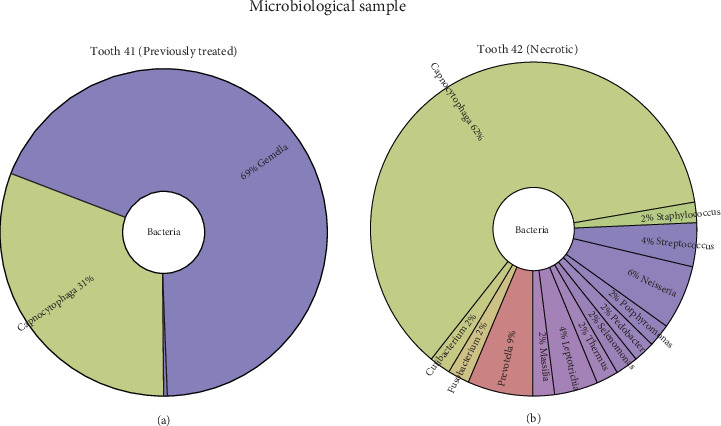
Microbiological profile of Teeth 41 and 42. Radial graphs representing the microbiological composition of Teeth 41 and 42 were obtained through polymerase chain reaction (PCR) amplification and sequencing of the 16S ribosomal DNA gene. (a) The microbiological profile of Tooth 41 shows a composition dominated by the genera *Gemella* and *Capnocytophaga*, with minor representation from other genera. (b) The microbiological profile of Tooth 42 shows a more complex bacterial community with a predominance of *Capnocytophaga* (62%), *Prevotella*, *Fusobacterium*, and *Porphyromonas*. Additional genera identified include *Veillonella*, *Leptotrichia*, and *Actinomyces*.

The radial graphs (Krona) of the microbial composition for Teeth 41 and 42 (Figure 5) highlight the distribution of various bacterial genera. Tooth 41 showed a simpler microbial composition, primarily consisting of *Gemella* and *Capnocytophaga* genera. On the other hand, Tooth 42 exhibited a more complex bacterial profile with a significant representation of *Capnocytophaga* (62%), *Prevotella*, and other bacteria. The presence of genera associated with periodontal and endodontic diseases, such as *Prevotella*, *Fusobacterium*, and *Porphyromonas*, was detected in variable abundance (from 2% to 6% of the total sample) only in Tooth 42.

## 3. Discussion

This case report shows the successful nonsurgical retreatment of a 16 × 9 mm osteolytic lesion, demonstrating favorable healing and buccal bone regeneration within 30 months. This approach minimizes patient discomfort and risks of complications associated with surgical procedures [1]. The decision for a nonsurgical approach was influenced by the intention to avoid surgical risks, the chief complaint, and the young age of the patient. The positive outcome achieved supports the viability of this conservative strategy in similar clinical situations.

The use of CBCT and its 3D reconstruction played an important role in this case. The advanced imaging allowed for an accurate assessment of the extent of the lesion, the degree of buccal bone destruction, and the proximity to critical anatomical structures. At the 6-month follow-up, the CBCT scan confirmed buccal bone regeneration, highlighting the efficacy of the treatment. The 3D reconstruction from CBCT imaging clearly shows the regeneration of the cortical bone, especially in the buccal bone area. One limitation of this case is the lack of histopathological confirmation of the periapical lesion. As the lesion was treated nonsurgically, no biopsy could be performed to definitively distinguish between a true apical cyst and a periapical granuloma. Although radiographic and CBCT findings suggested a large osteolytic lesion, these imaging modalities do not allow for a conclusive diagnosis. Therefore, in the absence of histological analysis, the nature of the lesion remains presumptive.

In this case report, a combination of techniques involving manual, rotary, and reciprocating instruments was used. The use of reciprocation was chosen due to its mechanical and clinical advantages. The reciprocating motion allows the effective removal of previous obturation materials and endodontic remnants while significantly reducing the risk of instrument fracture and mechanical stress on the canal walls [18, 19]. In this case, an apical enlargement was initially performed up to size 50.05 with Reciproc Blue. Then, a 60.02 K-file instrumentation was made to ensure optimal cleaning and shaping of the apical third of the canal. The apical third is well known to be a critical area in endodontic treatment due to its complex anatomy and the presence of apical deltas, lateral canals, and isthmuses [20, 21]. This final step is important for the complete removal of infected tissue and to allow deeper penetration of irrigants, thus enhancing the antimicrobial efficacy of the treatment [22, 23].

Incremental instrumentation for root canal enlargement, facilitated by reciprocating instruments, results in greater exposure and disinfection of dentinal tubules, which is important in retreatment cases involving persistent bacteria and biofilms. The exposure of dentinal tubules aids in the effective removal of microbial contamination, helping to improve the healing of large periapical lesions [24]. However, this procedure requires careful consideration to avoid excessive weakening of the root structure.

Although the complete elimination of bacteria from the root canal system is challenging due to the complex anatomy, the goal of endodontic treatment is to reduce the bacterial load to levels that allow periapical healing. Advanced techniques like NGS can help evaluate the effectiveness of retreatment procedures in achieving this goal. Understanding the specific bacterial profiles present in infected root canals can influence treatment strategies and outcomes.

The microbiological analysis, in this case, revealed the presence of *Gemella* and *Capnocytophaga* in Tooth 41 and a predominance of *Capnocytophaga* and *Prevotella* in Tooth 42. *Prevotella*, along with *Porphyromonas* and *Fusobacterium* genera, contains several periodontal and endodontic virulent species (e.g., *Porphyromonas gingivalis*, *Prevotella intermedia*, and *Fusobacterium nucleatum*) known to induce local inflammation [25] and periodontal and periapical bone loss through the activation of osteoclasts and RANKL bone resorption pathways [26].

In the present clinical case, the NGS analysis did not influence the clinical treatment plan, as it was not intended to guide therapeutic decisions. The main objective of including NGS was to investigate the bacterial composition of a large and complex periapical lesion involving multiple adjacent teeth. Identifying the specific microbial communities allowed us to better understand the diversity and complexity of the endodontic infection in this type of lesion. Although this information did not affect the clinical management, it provides valuable information for future research. Further studies may evaluate whether particular microbiome profiles are associated with delayed healing or persistent infection and whether tailored disinfection protocols or adjunctive therapies could be developed accordingly.

Even though the apical region of both teeth resulted in the same bone defect, the microbiological analysis revealed distinct bacterial profiles. The variation in bacterial populations between the two teeth suggests that local microenvironmental factors and previous treatments might influence microbial colonization, further complicating the infection dynamics within the same lesion.

Radiographic follow-ups (Figures 3b, 3c, 3d, 3e, 3f, 3g, and 3h) showed the apical extrusion and the morphological modification over time of the bioceramic sealer. The role and biological impact of these bioactive materials when extruded beyond the apex are still debated in the literature. Few clinical studies discuss and compare the outcomes of root canal treatments in this scenario [27–31]. Recent evidence also highlights the need for a more critical evaluation of this phenomenon. A systematic review and meta-analysis [7] showed that apical extrusion of bioceramic sealers does not appear to significantly increase postoperative pain or compromise treatment success, supporting their clinical safety and favorable biological response. In the present case report, throughout the follow-up period of 30 months, the extruded sealer remained visible in the periapical area. The radiographs clearly show the gradual integration of the bioceramic sealer into the surrounding tissues without producing adverse reactions or postoperative pain. This observation aligns with previous studies suggesting that bioceramic sealers are well tolerated by periapical tissues and can create a favorable biological environment that may contribute to bone healing and regeneration.

While the use of CeraSeal, a premixed bioceramic sealer, may have contributed to the healing process due to its bioactive properties, it is important to recognize that successful endodontic treatment outcomes are multifactorial. The meticulous cleaning and shaping of the root canal system, effective disinfection protocols, and proper obturation techniques all play critical roles. Although bioceramic sealers have been shown to promote osteogenesis and are biocompatible with periapical tissues [32, 33], attributing the healing solely to the sealer would be speculative. Further clinical studies are needed to determine the specific impact of bioceramic sealers on the healing of large periapical lesions.

A nonsurgical approach represents a less invasive technique with promising outcomes, as seen in this case. A conservative approach is aimed at treating the infection and promoting healing through debridement, disinfection, and the use of advanced sealing materials. While the current literature supports the use of bioceramic sealers, further research is warranted to explore their long-term durability and the mechanisms underlying their bioactivity. Additionally, more clinical trials are needed to establish standardized protocols for the nonsurgical management of large periapical lesions using bioceramic materials.

## 4. Conclusion

The nonsurgical management of a 16 × 9 mm periapical lesion shows the potential effectiveness of conservative endodontic retreatment in promoting the healing of extensive osteolytic lesions. The bone regeneration and complete healing observed over the 30-month follow-up suggest that meticulous cleaning and shaping of the root canal system, effective disinfection protocols, and proper obturation techniques are critical factors in achieving favorable outcomes. While the use of a premixed bioceramic sealer may have contributed to the healing process due to its bioactive properties, it is important to acknowledge that the positive result cannot be attributed solely to the sealer. Further clinical studies are necessary to determine the specific impact of bioceramic sealers on the healing of large periapical lesions. Additionally, the microbiological findings obtained through NGS provide valuable insights into the bacterial profiles associated with periapical infections, emphasizing the importance of understanding microbial communities in endodontic treatment. Although NGS analysis did not directly influence the clinical decision-making in this case, it provided a valuable microbial characterization of the root canal infections. This information may guide future personalized disinfection strategies and contribute to a better understanding of microbial dynamics in persistent lesions.

## Patient Perspective

From the beginning, the patient was collaborative and showed a positive attitude toward the proposed treatment. He was interested in every phase of the clinical procedure, actively attending all scheduled follow-ups. The patient expressed particular satisfaction in avoiding surgical intervention and, at the end of the treatment, reported being highly pleased with the overall outcome.

## Supporting Information

Supporting Information Additional supporting information can be found online in the Supporting Information section. Figure S1. PRICE 2020 checklist. This supporting information includes the completed PRICE 2020 checklist for case reports in endodontics, documenting adherence to the recommended reporting standards.
