# Supplementary material for: Nonsurgical Retreatment of a 16 × 9 mm Osteolytic Lesion With Buccal Bone Regeneration Using a Bioceramic Sealer: A Clinical and NGS-Based Microbiological Case Report With 30-Month Follow-Up
Source: Case Rep Dent. 2025 Oct 9;2025:9703223. doi: 10.1155/crid/9703223 (PMC12530936; doi:10.1155/crid/9703223)
Supplement: Supporting Information — Additional supporting information can be found online in the Supporting Information section. Figure S1. PRICE 2020 checklist. This supporting information includes the completed PRICE 2020 checklist for case reports in endodontics, documenting adherence to the recommended reporting standards. [file 9703223.f1.docx]

**PRICE 2020**

**Checklist of items to be included when reporting case reports in Endodontics***

| Section/Topic | Item number | Checklist Item | Reported on page number |
| --- | --- | --- | --- |
| **Title** | 1a | The words “case report(s)” must be included in the title | 1 |
|  | 1b | The area of interest (e.g. anatomy, disease, treatment) must be included briefly in the title | 1 |
| **Keywords** | 2a | At least two relevant keywords**,** preferably MeSH terms, related to the content of the case report must be included | 1 |
| **Abstract** | 3a | The Introduction must contain information on how the report is novel and contributes to the literature, clinical practice and/or fills a gap(s) in knowledge | 1 |
|  | 3b | The Body must describe the main clinical findings, including symptoms and signs, if present | 1 |
|  | 3c | The Body must describe the main radiographic/histological/ laboratory/diagnostic findings | 1 |
|  | 3d | The Body must describe the main outcomes of treatment, if active treatment has been provided | 1 |
|  | 3e | The Conclusion(s) must contain the main “take-away” lesson(s), sometimes referred to as key learning point(s) | 1 |
| **Introduction** | 4a | A background summary of the case(s) with relevant information must be provided | 2 |
| **Informed consent** | 5a | A clear statement that informed, valid consent was obtained from the patient(s) must be provided | 4 |
| **Case report information** | 6a | The age of the patient(s) must be provided | 4 |
|  | 6b | The gender of the patient(s) must be provided | 4 |
|  | 6c | The ethnicity of the patient(s) must be provided, if relevant |  |
|  | 6d | The main concern, chief complaint or symptoms of the patient(s), if any, must be provided | 4 |
|  | 6e | The medical history of the patient(s) must be provided, if relevant | 4 |
|  | 6f | The dental history of the patient(s) must be provided, if relevant | 4 |
|  | 6g | The family history of the patient if associated with the primary complaint must be provided, if relevant |  |
|  | 6h | The psychosocial history of the patient if associated with the primary complaint must be provided, if relevant |  |
|  | 6i | Genetic information, including details of relevant comorbidities and past interventions and their outcomes must be provided when possible, if relevant |  |
|  | 6j | Extra-oral findings must be provided, if relevant |  |
|  | 6k | General intra-oral findings must be provided when relevant, e.g. carious lesions, restorations, periodontal condition, soft tissues etc. |  |
|  | 6l | Important/relevant dates and times (in the text, or a table or figure) must be provided in chronological order | 4 |
|  | 6m | The diagnostic methods and the results for the specific tooth/teeth (e.g. pulp sensibility test, tenderness, mobility, periodontal probing depths, laboratory investigations, imaging techniques, or other special tests) must be provided | 4 |
|  | 6n | The diagnostic challenges, if any, must be provided | 4 |
|  | 6o | The diagnostic reasoning including other possible diagnoses that were considered must be provided | 4 |
|  | 6p | The active treatment (s) or intervention(s) performed, if any, must be provided | 4 |
|  | 6q | Any modifications to the proposed treatment(s) or intervention(s), if necessary, must be provided |  |
|  | 6r | The assessment method(s) used to determine the clinician-assessed and patient-assessed treatment outcomes and their results must be provided | 4 |
|  | 6s | Adverse and unanticipated events or consequences, if any, must be provided |  |
| **Discussion** | 7a | The specific treatment(s) and intervention(s) (if any) must be discussed with reference to the relevant literature | 7 |
|  | 7b | The strengths of the case report and its importance must be discussed with reference to the relevant literature | 7 |
|  | 7c | The limitations of the case report must be discussed | 7 |
|  | 7d | The rationale for the conclusion(s) must be discussed | 7 |
| **Patient perspective** | 8a | Feedback from the patient on the treatment and the care they received should be provided, if relevant |  |
| **Conclusion** | 9a | Explicit conclusion(s), i.e. the main “take-away” lessons must be provided | 9 |
|  | 9b | Implications for clinical practice or future research must be provided | 9 |
| **Funding details** | 10a | Sources of funding and other support (such as supply of instruments, equipment) as well as the role of funders must be acknowledged and described | 9 |
| **Conflict of interest** | 11a | An explicit statement on conflicts of interest must be provided | 9 |
| **Quality of images** | 12a | Details of the equipment, software and settings used to acquire the image(s) must be described in the text or legend | 4 |
|  | 12b | The reason why the image(s) was acquired and the rationale for its inclusion in the manuscript must be provided in the text | 4 |
|  | 12c | The circumstances (conditions) under which the image(s) were viewed and evaluated by the authors must be provided in the text | 4 |
|  | 12d | The resolution and any magnification of the image(s) or any modifications/enhancements (e.g. adjustments for brightness, colour balance, or magnification, image smoothing, staining etc.) that were carried out must be described in the text or legend |  |
|  | 12e | Patient(s) identifiers (names, patient numbers) must be removed to ensure they are anonymised |  |
|  | 12f | An interpretation of the findings (meaning and implications) from the image (s) must be provided in the text | 4 |
|  | 12g | The legend associated with each image must describe clearly what the subject is and what specific feature(s) it illustrates. Legends associated with images of patients must describe the age, gender and ethnicity of the person, if relevant | 10 |
|  | 12h | Markers/labels must be used to identify the key information in the image(s) and be defined in the legend or as a footnote |  |
|  | 12i | The legend of each image must include an explanation whether it is pre-treatment, intra-treatment or post-treatment and, if relevant, how images over time were standardised | 10 |

***From: Nagendrababu V, Chong BS, McCabe P, Shah PK, Priya E, Jayaraman J, Pulikkotil SJ, Setzer FC, Sunde PT, Dummer PMH (2020) PRICE 2020 Guidelines for reporting case reports in Endodontics: A consensus-based development. *International Endodontic Journal* doi: 10.1111/iej.13285.**

**For further details visit:** [**http://pride-endodonticguidelines.org/**](http://pride-endodonticguidelines.org/)**price/**
